# Individualized Cost-Effectiveness Analysis

**DOI:** 10.1371/journal.pmed.1001058

**Published:** 2011-07-12

**Authors:** John P. A. Ioannidis, Alan M. Garber

**Affiliations:** 1 Stanford Prevention Research Center, Department of Medicine and Department of Health Research and Policy, Stanford University School of Medicine, Stanford, California, United States of America; 2 Center for Health Policy and Center for Primary Care and Outcomes Research, Stanford University School of Medicine, Stanford, California, United States of America; 3 Veterans Affairs Palo Alto Health Care System, Stanford, California, United States of America

## Abstract

John Ioannidis and Alan Garber discuss how to use incremental cost-effectiveness ratios (ICER) and related metrics so they can be useful for decision-making at the individual level, whether used by clinicians or individual patients.

**Summary Points**

- Cost-effectiveness analyses typically express their principal results as incremental cost-effectiveness ratios (ICERs).
- ICERs are useful in making decisions for allocation of resources at a population level, but typical ICER measures have shortcomings when used for individual decisions.
- For the same ICER, the cost-effectiveness may vary among individuals because not everyone assigns the same priorities to specific outcomes, shares the same attitudes toward risk, or faces the same distribution of expected outcomes.
- ICER information can be enhanced by providing additional metrics that individualize cost-effectiveness analyses.
- These metrics include the per person net benefit and cost, subgroup ICER estimates for observed measured sources of heterogeneity, and distributions of outcomes and costs for unknown or unmeasured sources of heterogeneity.

The results of a typical cost-effectiveness (cost–utility) analysis are expressed by the incremental cost-effectiveness ratio (ICER) [1]: the money required to gain a quality-adjusted life year (QALY) (i.e., one year with best possible quality of life) at a population level. The ICER concept is valuable for choosing among diverse interventions competing for limited resources. It was developed primarily for societal or group-level decisions, such as allocations of a fixed governmental health budget, and consequently is particularly helpful for health authorities and other decision-makers who wish to prioritize resource allocation in health care to numerous interventions on diverse diseases across whole systems. The major contribution of ICER to inform population-level decisions is even more obvious today, as health care costs are escalating and rational choices need to be made on how to contain cost without compromising health outcomes at the societal level. However, conventional ICERs are population-level tools, and fail to take into account important inter-individual differences that might affect the value of a particular intervention. The choice that maximizes the population's health or has the best ICER overall is not always the same as the best choice for a specific individual. Moreover, the best choices may differ for different individuals. There is thus interest in how to modify the ICER concept for applications in individual decision-making [2],[3]. In this essay, we aim to contribute to the discussion on how to use ICER and related metrics in a way that would be more useful for decision-making at the individual level, whether used by clinicians or individual patients.

## The Concept of Individualized Cost-Effectiveness and Individual Choices

By convention, the numerator of the ICER is the difference in cost of care between compared interventions. The denominator is the corresponding difference in health outcomes (usually measured in QALYs). The denominator combines disparate kinds of information: diverse health benefits and harms are summed into a net health outcome measure.

As typically measured, the ICER appraises the average experience with an intervention. However, several investigators have pointed out that working with averages is not good enough. According to Kravitz et al. [4], “averages do not apply to everyone.” As is now well-recognized, averages are problematic for interpreting clinical evidence, e.g., the results of randomized trials, where treatment effects (benefits, but also harms) are often heterogeneous, i.e., different for different types of patients [5]–[7]. The same challenges arise when one considers cost-effectiveness and decision-making. After all, randomized trials and other clinical studies typically feed their data into decision and cost-effectiveness models. Moreover, the granularity of the cost and outcomes per patient may be important to convey, i.e., specifying separately the different outcomes, so that clinicians and patients may be better informed and able to make better choices.

Sometimes there is no compelling reason to distinguish different outcomes rather than using a summary measure, especially if all the outcomes are similar in severity and consequences. For example, consider two medicines that treat the common cold. One of them reduces the average duration of the cold by three days, while the other reduces the average duration by four days, but on average the second one also causes one day of headache as a side effect. As it happens, the headaches are no better or worse than having a cold. From the patient's point of view, both drugs decrease the duration of symptomatic illness by three days, with no meaningful difference between them.

However, lumping outcomes can be problematic when two interventions, even if they have identical mean effects, have very different distributions of outcomes. For example, suppose one intervention reduces the duration of the cold by an extra day relative to the other treatment, but on rare occasions it causes side effects that are either severe or prolonged, so the net effect on health is identical, on average. Then, some patients might reasonably decide that they would prefer the drug that is less effective most of the time, rather than risk the small possibility of becoming very ill if they were unlucky enough to experience the major side effect.

Heterogeneity in outcomes and costs across patients may stem from both observed (known) and unobserved (unknown or unmeasured) sources. The former arise from variation in well-known, validated, measured characteristics that predictably influence outcomes or costs. For example, the benefit of an antibiotic and the cost of treating an infection might be well known to vary with the severity of the infection or the sensitivity of the causative organism. Heterogeneity from unobservable sources essentially reflects the variation in outcomes and costs that occurs within a group of individuals with otherwise identical observable characteristics. This variation is due to chance or due to predictors that are not discovered yet or are not measured.

## Situations Where the Traditional ICER Alone Is Insufficient

Table 1 lists situations where even though the ICER is the same, the individualized cost-effectiveness (and the resulting choices) may vary. Note that this distinction between requirements for individual decision-making and population-level ICERs is not a reflection of the typical perspective question, i.e., whose costs should be considered in the analysis (the society's, the payer's, the insurer's, and so forth). We assume here a societal perspective or the perspective of the perfect insurer in the calculation of all ICER estimates, as is standard practice in cost-effectiveness analysis [8].

**Table 1:** Some situations where the traditional ICER does not necessarily suffice for individualized decision-making.

Situations	Reasons
**Same ICER, different individualized cost–utility**	
Different individuals with different conditions	Priority may be given to major benefits
Different interventions for the same condition	Variability in risk aversion
Different individuals with the same condition	Variability in outcomes experienced
**Same ICER, same individualized cost–utility, different choices**	
Similar individuals with the same condition	Variability in risk aversion
Same individual	Different background health
	Different personal circumstances

First, when the ICER is the same for two treatments for different conditions, each intervention may seem to be an equally good investment. However, a patient may have strong preferences for one over the other. For example, consider one intervention that improves outcomes in a few very sick patients who would die otherwise, but can live good-quality lives with treatment; both the benefits and the costs are very large. Another intervention has an equally favorable ICER, but it confers very small benefits to many individuals who are not very sick at baseline, with little cost per person. In many circumstances, the former intervention would be favored. Economists recognize that individuals place a greater value for the same absolute gains in life expectancy as life expectancy becomes shorter, all else being equal. For example, cadaveric donor liver transplantation has an ICER of US$42,000/QALY [9] and in many respects is more widely adopted than intensive lifestyle intervention in adults with impaired glucose tolerance, which has at least as favorable an ICER (one analysis gives even an ICER of US$11,000/QALY [10]). Most of the time, selecting between interventions for different conditions is a challenge that policy-makers, not individuals, face. However, individuals can face similar situations, particularly at different times in their lives.

Second, two different interventions for the same condition may have the same ICER, and the same expected outcomes, but the distributions of health outcomes might differ—one might have greater variance than the other. Some people may be more risk-averse than others [11]–[14]. Risk-averse people would sometimes prefer an intervention that provided inferior outcomes, on average, to an alternative that also had greater variance in outcomes. For example, suppose that two surgical interventions have the same ICER of $25,000/QALY. One operation costs $50,000 and confers 2.1 QALYs of benefit against 0.1 QALYs of harms due to a manageable problem that stems from the surgery. Another more aggressive procedure also costs $50,000 but confers 2.3 QALYs of benefit and, on average, 0.3 QALY of expected harm: 99% of the patients get no harm, but 1% die during the surgery and lose 30 years of life. Even though both operations have the same ICER, risk-averse patients may prefer to avoid the aggressive surgery.

Finally, the best choice may vary with an individual's background health and other, often highly personal, circumstances. Preferences over length of life may not be a simple function of its length. For example, a patient with late-stage metastatic cancer may wish to live until a landmark date, e.g., her daughter's wedding. The value of additional days of life may decline sharply after the landmark event. Among interventions with the same ICER, the patient will choose the one that maximizes the chances of remaining alive until the wedding.

These limitations of conventional cost-effectiveness analyses have long been recognized, yet the focus on ICER values averaged across a population is widespread. Effective communication of individualized analyses may require alternative approaches to presenting cost-effectiveness information.

## Possible Ways to Present Individualized Cost-Effectiveness

Box 1 lists different approaches to individualizing cost-effectiveness.

**Box 1. Approaches to Presenting Individualized Cost-Effectiveness Analysis**

- Per person benefit in QALYs or quality adjusted life days
- Per person cost
- Subgroup-specific estimates of per person benefit and per person cost for well-documented and validated subgroups based on known sources of heterogeneity
- Distribution (extent of variance) of benefits overall and in subgroup-specific estimates
- Distribution (extent of variance) in costs overall and in subgroup-specific estimates

### Per Person Benefit and Cost

The reporting of the expected cost and QALY gain or loss per person is already widely considered standard practice for a good cost-effectiveness analysis. However, even recent papers don't always report these numbers transparently. To illustrate how this works, suppose an analysis simulates costs and expected outcomes for 1,000,000 patients. Eventually, the analysis shows that 500 QALYs are gained by the new intervention versus a comparator at an incremental cost of $5,000,000. The ICER is $5,000,000/500 = $10,000/QALY. The per person cost is $5, for a gain of 0.0005 QALYs.

Table 2 shows the traditional ICER and calculated per person information from eight examples of recent cost-effectiveness analyses [15]–[22]. The individualized information is often easier to understand when the QALYs are expressed in days, i.e., in quality-adjusted life days (QALDs), because for many interventions the increase in QALYs is much less than one QALY. As shown, the absolute magnitudes of the net benefit and of the cost per person vary enormously across interventions, far more than the variation in the ICER. The average QALYs (or QALDs) gained per person varies from 0.18 days for screening for postnatal depression to 223 days for laparoscopic fundoplication for gasto-esophageal reflux, a more than 1,200-fold difference. The cost per person varies from about US$9 for rotavirus vaccination to US$2,586 for the fundoplication, an almost 300-fold difference. Conversely, in these examples, differences in traditional ICER are much smaller.

**Table 2:** Examples of ICERs and calculated per person benefits and costs.

Reference	Ratio Description	Traditional ICER (US Dollars/QALY)	Per Person Cost (US Dollars) and Benefit (QALDs)
Finckh et al. [15]	Early disease-modifying anti-rheumatic drug strategy versus pyramid strategy in adults with very early rheumatoid arthritis	$4,849	Pay $1,450 to gain 110 days
Eckman et al. [16]	Pharmacogenetic testing for personalized dosing of warfarin during induction versus no genotyping in newly diagnosed nonvalvular atrial fibrillation and no contraindications to warfarin	$171,750	Pay $369 to gain 0.78 days
Pletcher et al. [17]	Full adherence to Adult Treatment Panel III guidelines for lipid lowering versus current baseline adherence in ages 35–85 years	$42,000	Pay $328 to gain 2.83 days
Paulden et al. [18]	Routine screening for postnatal depression in primary care versus usual care in women assessed for postnatal depression, Edinburgh postnatal depression scale cutoff point = 16	$65,765	Pay $38.70 to gain 0.18 days
Latimer et al. [19]	Celecoxib (200 mg) plus proton pump inhibitor versus etoricoxib (30 mg) plus proton pump inhibitor in patients aged 55 years with osteoarthritis	$17,192	Pay $126.60 to gain 3.38 days
	Etoricoxib (30 mg) plus proton pump inhibitor versus diclofenac (100 mg) plus proton pump inhibitor in patients aged 55 years with osteoarthritis	$11,955	Pay $93 to gain 2.66 days
	Diclofenac (100 mg) plus proton pump inhibitor versus no treatment in patients aged 55 years with osteoarthritis	$11,142	Pay $31.70 to gain 1.04 days
Epstein et al. [20]	Laparoscopic surgery versus continued medical management in patients aged 45 and stable on gastro-esophageal reflux disease medication	$4,237	Pay $2,586 to gain 223 days
Barton et al. [22]	Dietary intervention plus strengthening exercises versus strengthening exercises in adults aged 45 with self-reported knee pain and BMI = 28	$11,256	Pay $642 to gain 20.8 days
	Dietary intervention plus strengthening exercises versus dietary intervention in adults aged 45 with self-reported knee pain and BMI = 28	−$13,702 (cost-saving)	Gain $192 and gain 5.11 days
	Dietary intervention plus strengthening exercises versus leaflet provision in adults aged 45 with self-reported knee pain and BMI = 28	$17,038	Pay $1,035 to gain 53.7 days
Rose et al. [21]	Vaccination with two doses of rotavirus vaccine versus no vaccination in India	$160	Pay $8.6 to gain 19.7 days

For cost-effectiveness using British pounds, a conversion of 1.6 US dollars per British pound has been used to convert values to US dollars. BMI, body mass index.

### Observed Sources of Heterogeneity

Average results can be misleading for many patients [23],[24]. Whenever possible, information should be tailored to identifiable subgroups with characteristics that explain variability in outcomes and costs. Costs and outcomes should be presented for groups with different absolute benefits (even if relative benefits, or relative risk reduction from an intervention, are identical). Frequently authors are justifiably reluctant to do so, particularly when the information is derived from post-hoc subgroup analyses of trial data. The extent of desirable subgrouping depends on the available evidence. There is a tipping point at which stratifying outcomes too finely and with limited evidence becomes cumbersome and introduces too much uncertainty to be useful for making decisions. However, when well-validated prognostic models are available or studies using sufficient independent data support the specific subgroup classifications [25],[26], cost–utility information can be confidently individualized by level of predicted risk and subgroup. For example, a cost-effectiveness analysis of tissue plasminogen activator versus streptokinase for thrombolysis in acute myocardial infarction has presented subgroup ICER estimates for different locations of infarction and different age groups, with up to 16-fold differences across these subgroups [27].

### Unobserved Sources of Heterogeneity

When heterogeneity in outcomes or costs arises from unknown sources, every patient who receives an intervention initially faces the same distribution of potential outcomes. When heterogeneity matters, information on the distribution of potential outcomes and costs should also be presented, even though we can't predict which patient will get the most benefit, and who will get the rare but devastating adverse event. An illustrative example of how to present this information appears in Table 3.

**Table 3:** Hypothetical example of different individual experiences for patients who do and do not experience different events (benefit, harm, major harm, or other rare events).

Category of Patient Experience	Percent of Patients	QALDs for Benefits/Harms	Cost ( Dollars)	Per Person Cost (Dollars) and Benefit (QALDs)
No benefit, no harm	80%	0/0	$10	Pay $10 for no gain
Benefit, no harm	10%	30/0	$8	Pay $8 to gain 30 days
No benefit, harm	6%	0/5	$100	Pay $100 and lose 5 days
No benefit, major harm	2%	0/50	$1,000	Pay $1,000 and lose 50 days
Benefit, harm	0.75%	30/5	$80	Pay $80 to gain 25 days
Benefit, major harm	0.25%	30/50	$800	Pay $800 and lose 20 days
All other categories with rare events	1%	Varies per category, average 5/10	Varies per category, average $260	Varies per category, on average pay $260 and lose 5 days
**Average**		3.35/1.56	$40	Pay $40 to gain 1.79 days

In the specific example, there are no known predictors that identify the patients who will experience any of these events. The intervention of interest confers a benefit in one out of nine treated people, a minor harm in 6.8%, a major harm in 2.3%, and other events in 1%. On average, it costs $40 to gain 1.79 days. However, for the large majority of patients (80%) who will experience no events, good or bad, the cost is $10 and there is no gain (or loss) at all. For patients in the 10% who will get the benefit, without any harm, one has to pay only $8 per patient and each patient gains 30 days. Conversely, for patients in the 2% who experience the major harm and not the benefit, one has to pay $1,000 per patient and each patient loses 50 days.

The optimal way to present distributions depends on the type of variable. For example, most continuous measures (including cost) can be summarized with means/medians and standard deviations or interquartile ranges. Time-to-event outcomes such as long-term survival are conveniently represented by Kaplan-Meier plots. Utility or quality weights can also be plotted over time, with expected mean values and variances thereof. Presenting distributions in full detail requires space, and their interpretation can be burdensome, but both problems can be addressed, e.g., by online supplements that include detailed explanatory material.

## Societal versus Patient Perspective

Thus far we have assumed a societal perspective for the calculation of costs. We should acknowledge that if patients don't have to pay for any of the health care costs themselves, they may opt for the choice that maximizes their net benefits regardless of the cost. However, in many health systems, cost is taken into account and limits the choices of individuals.

Even in countries with national health systems that have minimized the role of individual cost-sharing, individuals pay growing shares of the costs of health and health-related services out-of-pocket. Direct-to-consumer advertisement, over-the-counter interventions, and personalized (e.g., genomic) testing are examples of growing markets that try to appeal to individuals who pay for services with their own funds [28]–[30]. Because patients spending their own money have a compelling interest in the value of the services they consume, cost-effectiveness analysis is not solely the province of government or corporate decision-makers. Whenever costs and outcomes from the patient perspective differ from those from the population/societal perspective, costs and outcomes should be reported from both perspectives. This will greatly increase the range of usefulness of the analysis. Across different fields of economics, consumer demand is an important consideration. It is possible that policy-makers use ICER to make decisions currently without information that patients would find useful.

Moreover, cost-effectiveness analysis has relevance to patients, even if they do not bear costs directly, and to physicians with an interest in ensuring the wise use of resources. The costs of care are typically hidden or unknown to patients and physicians. Similarly, there may be a poor understanding of what outcomes are really at stake for an individual. Further studies are needed to clarify whether provision of individualized information influences the behavior and choices of physicians and patients.

## Caveats

Once cost, benefits, and harms are presented separately for different types of patients, one can take an extra step and directly incorporate personal preferences in the calculations, i.e., placing different weights on the same side effects and benefits of therapy for different patients [31],[32]. One may even assign different values to different time sequences of beneficial and harmful events, e.g., by reporting the healthy-years equivalents of lifetime health profiles [33]–[35]. However, personal weights for each type of cost and outcome and healthy-years equivalents for each of many possible complex lifetime health profiles are often difficult to elicit.

Individualized analysis is not always necessary or appropriate. First, the individualized cost-effectiveness information is directly meaningful only for interventions and strategies that are applied to a single person and where the beneficial or harmful impacts of the intervention do not extend beyond the specific type of individual. For example, full individualization cannot be applied to a cost-effectiveness analysis of modernization of adult critical care services [36], where the intervention is made at the level of a population service. Also, in cost-effectiveness analyses of vaccination of boys with human papilloma virus vaccine [37] or of influenza vaccination strategies [38], the intended benefits extend also to the partners or contacts, respectively, of the vaccinated individuals. In addition to the personal stake for the vaccinated individuals, implications for the health of other people may be substantial. Sometimes, the cumulative impact on other people's health may far exceed the impact on the single individual who gets such interventions.

Furthermore, estimates of individualized cost-effectiveness, much like traditional ICER estimates, are subject to errors in the assumptions that feed into their calculations. These errors may stem from a lack of evidence, poor representation of the evidence, or even manipulations due to conflicts of interest [39]–[41]. Erroneous inferences may result from unrealistic modeling of the decision process or unrealistic estimates for the various benefits, harms, and costs. Moreover, uncertainty in these values [42] or even in risk aversion [43] should not be underestimated. Sensitivity analyses incorporating a range of values for key parameters can be applied to the individualized cost-effectiveness, much as they are applied to the traditional ICER.

## Concluding Comments

Cost-effectiveness analysis offers a foundation for rational decision-making and can be very helpful in making health care more efficient and effective at the population level [44]. Such analyses can often be more useful for clinicians and for individual patients as well, when they individualize the cost–utility information they present. Individual-tailored information can complement the traditional ICER.

## Abbreviations

ICER: incremental cost-effectiveness ratio
QALD: quality-adjusted life day
QALY: quality-adjusted life year

## References

[1] Gold MR, Siegel JE, Russell LB, Weinstein MC (1996). Cost-effectiveness in health and medicine.

[2] Willan AR (2001). On the probability of cost-effectiveness using data from randomized clinical trials.. BMC Med Res Methodol.

[3] O'Hagan A, Stevens JW (2002). The probability of cost-effectiveness.. BMC Med Res Methodol.

[4] Kravitz RL, Duan N, Braslow J (2004). Evidence-based medicine, heterogeneity of treatment effects, and the trouble with averages.. Milbank Q.

[5] McLaughlin MJ, HTE Policy Roundtable Panel (2007). Healthcare policy implications of heterogeneity of treatment effects.. Am J Med.

[6] Greenfield S, Kravitz R, Duan N, Kaplan SH (2007). Heterogeneity of treatment effects: implications for guidelines, payment, and quality assessment.. Am J Med.

[7] Glasziou PP, Irwig LM (1995). An evidence based approach to individualising treatment.. BMJ.

[8] Garber AM, Culyer AJ, Newhouse JP (2000). Advances in cost-effectiveness analysis of health interventions.. Handbook of health economics, Volume 1.

[9] Mendeloff J, Ko K, Roberts MS, Byrne M, Dew MA (2004). Procuring organ donors as a health investment: how much should we be willing to spend?. Transplanation.

[10] Herman WH, Hoerger TJ, Brandle M, Hicks K, Sorensen S (2005). The cost-effectiveness of lifestyle modification or metformin in preventing type 2 diabetes in adults with impaired glucose tolerance.. Ann Intern Med.

[11] Luce R (2000). Utility of gains and losses: measurement-theoretical and experimental approaches.

[12] Byrnes JP, Miller DC, Schafer WD (1999). Gender differences in risk taking: A meta-analysis.. Psych Bull.

[13] Elbasha EH (2005). Risk aversion and uncertainty in cost-effectiveness analysis: the expected-utility, moment-generating function approach.. Health Econ.

[14] Sapienza P, Zingales L, Maestripieri D (2009). Gender differences in financial risk aversion and career choices are affected by testosterone.. Proc Natl Acad Sci U S A.

[15] Finckh A, Bansback N, Marra CA, Anis AH, Michaud K (2009). Treatment of very early rheumatoid arthritis with symptomatic therapy, disease-modifying antirheumatic drugs, or biologic agents: a cost-effectiveness analysis.. Ann Intern Med.

[16] Eckman MH, Rosand J, Greenberg SM, Gage BF (2009). Cost-effectiveness of using pharmacogenetic information in warfarin dosing for patients with nonvalvular atrial fibrillation.. Ann Intern Med.

[17] Pletcher MJ, Lazar L, Bibbins-domingo K, Moran A, Rodondi N (2009). Comparing impact and cost-effectiveness of primary prevention strategies for lipid lowering.. Ann Intern Med.

[18] Paulden M, Palmer S, Hewitt C, Gilbody S (2009). Screening for postnatal depression in primary care: cost effectiveness analysis.. BMJ.

[19] Latimer N, Lord J, Grant RL, O'Mahony R, Dickson J (2009). Cost effectiveness of COX 2 selective inhibitors and traditional NSAIDs alone or in combination with a proton pump inhibitor for people with osteoarthritis.. BMJ.

[20] Epstein D, Bojke L, Sculpher MJ, REFLUX Trial Group (2009). Laparoscopic fundoplication compared with medical management for gastro-oesophageal reflux disease: cost effectiveness study.. BMJ.

[21] Rose J, Hawthorn RL, Watts B, Singer ME (2009). Public health impact and cost effectiveness of mass vaccination with live attenuated human rotavirus vaccine (RIX4414) in India: model based analysis.. BMJ.

[22] Barton GR, Sach TH, Jenkinson C, Doherty M, Avery AJ (2009). Lifestyle interventions for knee pain in overweight and obese adults aged > or  = 45: economic evaluation of randomised controlled trial.. BMJ.

[23] Laska EM, Meisner M, Siegel C (1997). Statistical inference for cost-effectiveness ratios.. Health Econ.

[24] Briggs A, Fenn P (1997). Trying to do better than average: a commentary on ‘statistical inference for cost-effectiveness ratios’.. Health Econ.

[25] Ioannidis JP, Lau J (1997). The impact of high-risk patients on the results of clinical trials.. J Clin Epidemiol.

[26] Kent DM, Rothwell PM, Ioannidis JP, Altman DG, Hayward RA (2010). Assessing and reporting heterogeneity in treatment effects in clinical trials: a proposal.. Trials.

[27] Mark DB, Hlatky MA, Califf RM, Naylor CD, Lee KL (1995). Cost effectiveness of thrombolytic therapy with tissue plasminogen activator as compared with streptokinase for acute myocardial infarction.. N Engl J Med.

[28] Gilbody S, Wilson P, Watt I (2005). Benefits and harms of direct to consumer advertising: a systematic review.. Qual Saf Health Care.

[29] Wright CF, Hall A, Zimmern RL (2011). Regulating direct-to-consumer genetic tests: What is all the fuss about?. Genet Med.

[30] Cohen JP, Paquette C, Cairns CP (2005). Switching prescription drugs to over the counter.. BMJ.

[31] Nease RF, Owens DK (1994). A method for estimating the cost-effectiveness of incorporating patient preferences into practice guidelines.. Med Decis Making.

[32] Gage BF, Cardinalli AB, Owens DK (1998). Cost-effectiveness of preference-based antithrombotic therapy for patients with nonvalvular atrial fibrillation.. Stroke.

[33] Gafni A, Birch S, Mehrez A (1993). Economics, health and health economics: HYEs (healthy-years equivalent) versus QALYs (quality-adjusted live-year).. J Health Econ.

[34] Mehrez A, Gafni A (1989). Quality-adjusted life years, utility theory, and healthy-years equivalents.. Med Decis Making.

[35] Mehrez A, Gafni A (1991). The healthy-years equivalents: how to measure them using the standard gamble approach..

[36] Hutchings A, Durand MA, Grieve R, Harrison D, Rowan K (2009). Evaluation of modernisation of adult critical care services in England: time series and cost effectiveness analysis.. BMJ.

[37] Kim JJ, Goldie SJ (2009). Cost effectiveness analysis of including boys in a human papillomavirus vaccination programme in the United States.. BMJ.

[38] Khazeni N, Hutton DW, Garber AM, Hupert N, Owens DK (2009). Effectiveness and cost-effectiveness of vaccination against pandemic influenza (H1N1) 2009.. Ann Intern Med.

[39] Bell CM, Urbach DR, Ray JG, Bayoumi A, Rosen AB (2006). Bias in published cost effectiveness studies: systematic review.. BMJ.

[40] Miners AH, Garau M, Fidan D, Fischer AJ (2005). Comparing estimates of cost effectiveness submitted to the National Institute for Clinical Excellence (NICE) by different organisations: retrospective study.. BMJ.

[41] Polyzos NP, Valachis A, Mauri D, Ioannidis JPA (2011). Sponsorship bias in assumptions of cost-effectiveness analysis: the diagnostic accuracy of the Pap test.. CMAJ.

[42] Hoch JS, Dewa CS (2007). Lessons from trial-based cost-effectiveness analyses of mental health interventions: why uncertainty about the outcome, estimate and willingness to pay matters.. Pharmacoeconomics.

[43] O'Brien BJ, Sculpher MJ (2000). Building uncertainty into cost-effectiveness rankings: portfolio risk-return tradeoffs and implications for decision rules.. Med Care.

[44] Owens DK, Qaseem A, Chou R, Shekelle P, Clinical Guidelines Committee of the American College of Physicians (2011). High-value, cost-conscious health care: concepts for clinicians to evaluate the benefits, harms, and costs of medical interventions.. Ann Intern Med.

